# Diagnostic, Clinical and Post-SARS-CoV-2 Scenarios in Cancer Patients with SARS-CoV-2: Retrospective Analysis in Three German Cancer Centers

**DOI:** 10.3390/cancers13122917

**Published:** 2021-06-11

**Authors:** Evgenii Shumilov, Petra Hoffknecht, Raphael Koch, Rudolf Peceny, Steffen Voigt, Nicole Schmidt, Micha Peeck, Ulrike Bacher, Simone Scheithauer, Lorenz Trümper, Georg Lenz, Andrea Kerkhoff, Annalen Bleckmann

**Affiliations:** 1 Department of Hematology and Medical Oncology, University Medicine Göttingen (UMG), 37075 Göttingen, Germany; raphael.koch@med.uni-goettingen.de (R.K.); nicole.schmidt@med.uni-goettingen.de (N.S.); veraulrike.bacher@insel.ch (U.B.); lorenz.truemper@med.uni-goettingen.de (L.T.); 2 Department of Thorax Oncology, Franziskus-Hospital Harderberg, Niels-Stensen-Kliniken, 49124 Georgsmarienhütte, Germany; petra.hoffknecht@niels-stensen-kliniken.de (P.H.); steffen.voigt@niels-stensen-kliniken.de (S.V.); 3 Department of Medical Oncology and Hematology, Franziskus-Hospital Harderberg, Niels-Stensen-Kliniken, 41924 Georgsmarienhütte, Germany; rudolf.peceny@niels-stensen-kliniken.de; 4 Medical Clinic A, Haematology, Haemostasiology, Oncology and Pulmonology, University Hospital Münster, 48149 Münster, Germany; micha.peeck@ukmuenster.de (M.P.); georg.lenz@ukmuenster.de (G.L.); andrea.kerkhoff@ukmuenster.de (A.K.); 5 Department of Hematology and Central Hematology Laboratory, Inselspital, Bern University Hospital, University of Bern, 3010 Bern, Switzerland; 6 Institute of Infection Control and Infectious Diseases, University Medicine Göttingen (UMG), 37075 Göttingen, Germany; simone.scheithauer@med.uni-goettingen.de

**Keywords:** SARS-CoV-2, COVID-19, cancer patients, SARS-CoV-2 scenarios

## Abstract

**Simple Summary:**

The study investigated diagnostic, clinical and post-SARS-CoV-2 scenarios in cancer patients with SARS-CoV-2 aiming to improve management of SARS-CoV-2 infections and cancer afterwards. Around half of patients were initially asymptomatic and were diagnosed with SARS-CoV-2 during routine or contact tracing screening. Of them, 33% developed COVID-19 lately. Eventually, predominant part of patients had asymptomatic SARS-CoV-2 or mild COVID-19 course. Lymphocytopenia preceding SARS-CoV-2 was associated with a significantly increased risk for severe or critical COVID-19 course. Commonly patients experienced a treatment delay post-SARS-CoV-2; one fifth developed progressive disease (PD) within that time and/or had to undergo therapy modifications following deterioration of the performance status or PD post-COVID-19. This study provides knowledge of real-life clinical courses of SARS-CoV-2 in oncology and contributes to improving therapeutic strategies for cancer patients in the COVID-19 pandemic.

**Abstract:**

Oncologists face challenges in the management of SARS-CoV-2 infections and post-SARS-CoV-2 cancer treatment. We analyzed diagnostic, clinical and post-SARS-CoV-2 scenarios in patients from three German cancer centers with RT-PCR confirmed SARS-CoV-2 infection. Sixty-three patients with SARS-CoV-2 and hematologic or solid neoplasms were included. Thirty patients were initially asymptomatic, 10 of whom developed COVID-19 symptoms subsequently. Altogether 20 (32%) patients were asymptomatic, 18 (29%) had mild, 12 (19%) severe and 13 (20%) critical courses. Lymphocytopenia increased risk of severe/critical COVID-19 three-fold (*p* = 0.015). Asymptomatic course was not associated with age, remission status, therapies or co-morbidities. Secondary bacterial infection accompanied more than one third of critical COVID-19 cases. Treatment was delayed post-SARS-CoV-2 in 46 patients, 9 of whom developed progressive disease (PD). Cancer therapy was modified in 8 SARS-CoV-2 survivors because of deteriorating performance or PD. At the last follow-up, 17 patients had died from COVID-19 (*n* = 8) or PD (*n* = 9) giving an estimated 73% four-month overall survival rate. SARS-CoV-2 infection has a heterogenous course in cancer patients. Lymphocytopenia carries a significant risk of severe/critical COVID-19. SARS-CoV-2 disruption of therapy is as serious as SARS-CoV-2 infection itself. Careful surveillance will allow early restart of the anti-cancer treatment.

## 1. Introduction

Declared a pandemic by the WHO in March 2020 corona virus disease 2019 (COVID-19) caused by the novel coronavirus 2019 (SARS-CoV-2) continues to pose many new challenges to the medical community worldwide. In view of the high contagiosity of the disease, as well as of its increased propensity for causing severe symptoms, patients with a compromised immune system belong to the most relevant risk group [1]. Due to the immunosuppressive effects of systemic anticancer therapy, cancer patients are one of the most vulnerable groups for COVID-19. With around 17 million new cancer cases diagnosed worldwide in 2018 [2] alone, the magnitude of possible SARS-CoV-2 consequences in the field of oncology can be very intimidating. Along these lines, several studies have documented a significantly higher risk for severe COVID-19 events among cancer patients compared with non-cancer individuals [3,4,5,6,7,8,9,10]. The UK Coronavirus Cancer Monitoring Project and the collaboration of 19 European cancer centers recently reported death rates of 28% and 33.6% in 800 and 890 oncological patients with COVID-19, respectively. This is dramatically higher than in the general population. Interestingly, age, gender and comorbidities were significantly associated with the lethality rate in both studies, whereas ongoing chemotherapy, targeted or immunotherapy, and the time point of its application did not worsen mortality [11,12]. Hematologists and oncologists must therefore carefully balance the tremendous risk of severe SARS-CoV-2 complications against the need to continue the cancer treatment, as the latter is automatically put on hold until recovery from COVID-19. Thus, questions such as the appropriate time to restart the cancer treatment, the duration of contagiousness, as well as about persistence of immune memory after COVID-19 and the risk of SARS-CoV-2 re-infection under systemic anticancer therapy will arise in virtually every case. Therefore, the knowledge of real-life clinical courses of SARS-CoV-2 infections, as well as of its diagnostic and therapeutic pitfalls in cancer patients can provide a basis for optimal decision-making strategies. Even though many efforts have been made to highlight an increased risk for cancer patients in the COVID-19 era, practice-oriented clinical analyses of SARS-CoV-2 infections and post-SARS-CoV-2 follow-up are underrepresented. Aiming to fill this gap, we analyzed the diagnostic and therapeutic strategies with regard to SARS-CoV-2 infections, as well as impact of the infection on the cancer course and treatment algorithms of 63 hematological and oncological patients from three German cancer centers.

## 2. Materials and Methods

### 2.1. Patients

Between March 2020 and February 2021, all patients with active or non-active cancer and a SARS-CoV-2 infection confirmed by real-time reverse transcriptase polymerase chain reaction (RT-PCR) presenting in the University Hospital of Göttingen, the University Hospital of Münster or the Departments of Thoracic Oncology and Medical Oncology and Hematology of the Franziskus-Hospital Harderberg, Georgsmarienhütte, Germany, were enrolled in the study. Both, hospitalized as well as outpatients with a SARS-CoV-2 infection with any cancer entity were analyzed. The retrospective data analysis was approved by decisions of the local ethics committees (Ethics Committee of the University Medical Center Göttingen No 31/10/20; Ethics Committee of the Lower Saxony Medical Association No 31/10/20; Ethics Committee of the Westphalia-Lippe Medical Association No 2020-955-b-S).

### 2.2. Methods

The diagnosis of SARS-CoV-2 was made by RT-PCR on nasopharyngeal and oropharyngeal swab material. Following commercial SARS-CoV-2 RT-PCR test systems were used: SARS-CoV-2 Cobas^®^ 6800/8800 (Roche, Basel, Switzerland), Alinity m SARS-CoV-2 assay (Abbott, Chicago, IL, USA), Xpert^®^ Xpress SARS-CoV-2 (Cepheid, Sunnyvale, CA, USA) and Genesic SARS-CoV-2 (G Healthcare, Chicago, IL, USA). Antibody response to SARS-CoV-2 was evaluated by enzyme-linked immunosorbent assays (ELISA) with Euroimmun (Lübeck, Germany) (1. Generation, IgA/IgG) or Abbott (Chicago, IL, USA) (IgG) kits.

### 2.3. Definitions

Patients with active cancer were defined as those with recurrent, regionally advanced or metastatic cancer for which treatment had been administered within six months in any setting (curative, palliative, radical, adjuvant or neoadjuvant). Patients with non-active cancer were defined as cancer survivors who did meet the above-mentioned criteria and were undergoing follow-up surveillance at the time of the SARS-CoV-2 infection. The COVID-19 severity categories were determined according to WHO guidelines: asymptomatic, mild (i.e., general symptoms, outpatient care), severe (i.e., hospital admission with need for oxygen supplementation) and critical (i.e., need for life support therapy) [13]. Seroconversion was defined as a transition of the test results for immunoglobulin G (IgG) or immunoglobulin A (IgA) against SARS-CoV-2 from negative to positive in sequential samples. Lymphocytopenia was documented when a lymphocyte count was below 1.0 × 10^9^/L. Routine SARS-CoV-2 screening was referred to in- and outpatients subjected randomly to PCR-testing without exhibiting any signs or symptoms of COVID-19. Contract tracing was defined as PCR-testing performed for patients with the history of close contacts to individuals with laboratory-confirmed or probable COVID-19. The post-SARS-CoV-2 follow-up implied monitoring of patients from the first positive SARS-CoV-2 RT-PCR result.

### 2.4. Analyzed Data

The patients were analyzed with regard to (A) demographics and cancer data including the last cancer therapy and remission status, as well as comorbidities at the time of first RT-PCR SARS-CoV-2 positive result, (B) the course of the SARS-CoV-2 infection including symptoms, severity and duration as well as laboratory and radiological findings, and finally (C) the cancer course in the post-SARS-CoV-2 follow-up. To this end, the impact of SARS-CoV-2 on overall survival, cancer therapy and remission status was evaluated. Patients were assessed for the presence of common comorbidities such as cardiovascular diseases (arterial hypertension, coronary artery disease, chronic heart failure, atrial fibrillation), metabolic diseases (diabetes mellitus, obesity), chronic respiratory and liver diseases, renal insufficiency, cerebrovascular diseases and autoimmune disorders.

### 2.5. Statistics

Categorical variables were summarized as frequencies and percentages, and continuous variables were summarized as medians and ranges. Probability of overall survival (OS) was calculated using the Kaplan-Meier method. Cumulative incidence curves were used for COVID-19 and cancer mortality in a competing risk. The Chi-square test of independence was used to determine significance of relationship between lymphocytopenia, histologic type of cancer (hematologic vs. solid), age and severity of COVID-19 course. The association between clinical characteristics of cancer patients and the occurrence of asymptomatic SARS-CoV-2 infection was assessed by Spearman’s correlation. A *p*-value less than 0.05 was considered statistically significant. Statistical analyses were performed with SPSS, version 26.0 (SPSS, Chicago, IL, USA) and R, version 3.6.2 (RStudio, Boston, MA, USA) (https://www.R-project.org/ (accessed on 25 February 2020)).

## 3. Results

### 3.1. Characteristics of Cancer Patients Positive for SARS-CoV-2

Sixty-three patients tested positive for SARS-CoV-2 were included in this study. Patient and disease characteristics at the time of the confirmed SARS-CoV-2 test are presented in Table 1. The median age was 62 (range, 19–85) with a slight predominance of males (35 males: 28 females). Hematologic and solid neoplasms were equally represented (32 vs. 31) with lymphomas the more frequent hematologic, and lung, breast or gastrointestinal tumors the most frequent solid neoplasms (Table 1 for details). In this case, 56 patients (89%) were categorized as active cancer cases, while the remaining seven were under follow-up surveillance after systemic cancer treatment. For details of the therapeutic modalities see Table 1. Particularly, 17 patients (30%) underwent immunotherapy-based treatment (either as monotherapy or combined with cytostatic agents or radiotherapy) presented predominantly by anti-CD20 antibodies (*n* = 9/17) and followed by programmed death-1/programmed death-ligand 1 (PD-1/PD-L1) (*n* = 6/17) and anti-CD38 antibodies (*n* = 1/17), as well as chimeric antigen receptor (CAR) T-cell therapy (*n* = 1/17). Before testing positive for SARS-CoV-2, thirty patients had received one therapy line (48%) and 2 (3%) received no therapy yet, whereas the remaining had undergone up to four or more therapy lines (Table 1). At the time of study enrollment 12 patients were in complete remission (CR), 12 in partial remission (PR), 11 had a stable disease (SD) and 20 had relapsed/progressive disease. Remission status had not yet been assessed in eight patients because treatment begin, or cancer diagnosis were so recent. Eighteen patients had no relevant comorbidity, while the others had up to four or more pre-existing conditions (Table 1).

### 3.2. Clinical, Laboratory and Imaging Findings in 63 Cancer Patients with SARS-CoV-2

**Figure 1:**
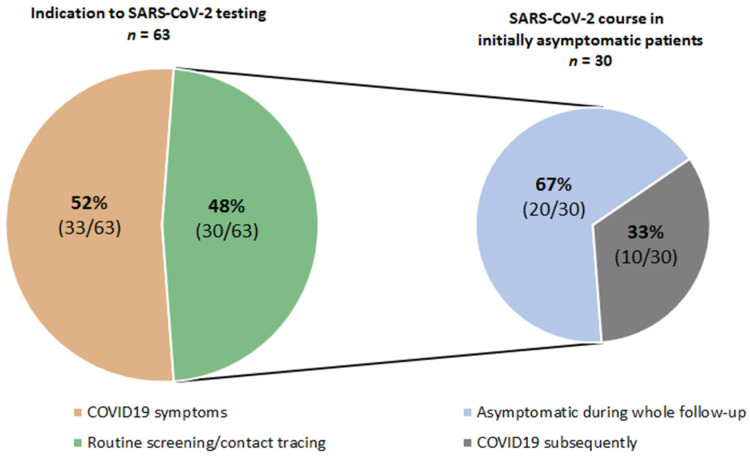
Indication for SARS-CoV-2 testing.

The laboratory, imaging and clinical findings of the patients are given in Table 2. The time from the last cancer treatment varied markedly from zero to nearly three years with a median of 13 days (Table 2). Approximately half of the patients exhibited COVID-19 symptoms primarily, while remaining patients were asymptomatic and tested within routine or contact tracing screening. A third of the initially asymptomatic patients subsequently developed typical COVID-19 symptoms giving a total of 43 patients with COVID-19 (Table 2, Figure 1). The median values for white blood cell count were in the normal range, while those for procalcitonin, C-reactive protein and lactate dehydrogenase (LDH) were elevated but there was a large spread in all parameters. Forty-six percent of the patients with an available differential blood count (*n* = 17/37) had lymphocytopenia (see Table 2). Even though immunophenotyping data was not available at the time point of SARS-CoV-2 diagnosis, B-cell depletion could be suggested in at least 35% (*n* = 6/17) of these patients who underwent either anti-CD20 immunotherapy (*n* = 5/6) or induction therapy within the GMALL (German Multicenter Study Group for Adult Acute Lymphoblastic Leukemia) protocol (*n* = 1/6) before. Chest imaging was documented as having been performed in 39 patients, and evidence of pneumonia was detected in 23 (59%) of them. Seroconversion with detectable IgA and/or IgG was seen in ten (63%) of the 16 patients who were tested for this with a median of 13 days. In six out of 43 COVID-19 cases (14%) secondary bacterial infection was confirmed subsequently. In four of them at least two microorganisms have been detected simultaneously, thereof two had the same and two different species, respectively. Bacteria could be cultivated from the following specimen: trachealbronchial fluid (*n* = 4/6), blood culture (*n* = 3/6) and urine (*n* = 2/6). The causative microorganisms were presented predominantly (*n* = 5/6) by Gram-negative bacteria: *Klebsiella pneumoniae* (*n* = 4/6), in one case together with *Klebsiella oxytoca*, *Escherichia coli* (*n* = 2/6) and *Pseudomonas aeruginosa* (*n* = 1/6). The remaining patient had evidence of *Staphylococcus aureus* in trachealbronchial fluid. Of note, five of six patients with secondary bacterial infection had critical COVID-19 compromising 38% (*n* = 5/13) of all critical cases. The remaining patient had severe COVID-19 (8%, *n* = 1/12).

### 3.3. Course of the SARS-CoV-2 Infection

**Figure 2:**
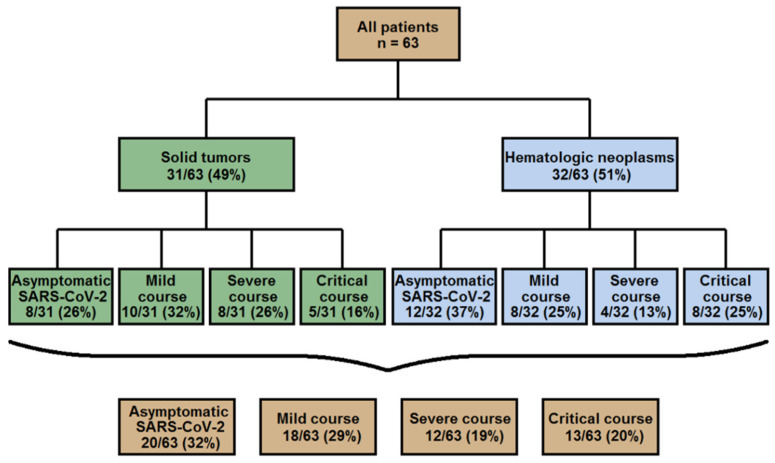
Distribution of SARS-CoV-2 infection severity.

Data on the course of the SARS-CoV-2 infection are presented in Table 3 and Figure 2. As mentioned above, 43 (68%) of the patients had COVID-19 symptoms, whereas 20 (32%) had an asymptomatic course.

#### 3.3.1. Asymptomatic SARS-CoV-2 Patients

Details of asymptomatic SARS-CoV-2 patients are listed in Table S1. Asymptomatic cases were seen among patients with hematologic 60% (*n* = 12/20) or solid 40% (*n* = 8/20) malignancies (*p* = 0.379). Analyzing the whole cohort of these patients, the occurrence of an asymptomatic course did not correlate with age (range, 21–81) (*p* = 0.328), the number of comorbidities (*p* = 0.816), therapy lines (*p* = 0.783) or level of remission (*p* = 0.718). Lymphoid neoplasms (83%, 10/12) were predominant among the hematologic malignancies: lymphomas (7/10), acute lymphatic leukemias (2/10) and multiple myeloma (1/10). Gastrointestinal cancer (4/8) was the most common malignancy among solid tumors followed by sarcomas (2/8), and breast and lung cancer (each 1/8). Of 20 patients, only three (15%) were under follow-up surveillance at the time point of SARS-CoV-2 diagnosis. The remaining patients were undergoing active cancer treatment with a broad spectrum of intensity and action principles. Particularly, cases with high intensity treatment (Table S1), such as advanced Hodgkin lymphoma in early post-transplant aplasia phase following high-dose chemotherapy/autologous stem cell transplantation (HDCT/ASCT) (pt #5), acute lymphatic leukemia under induction therapy with GMALL protocol (pt #10), pancreatic cancer under FOLFIRINOX (leucovorin, fluorouracil, irinotecan, oxaliplatin) (pt #15) or sarcoma (pt #20) under doxorubicin/ifosfamide regimen were documented. Regarding all patients with hematologic malignancies (*n* = 32) and comparing asymptomatic cases (*n* = 12) with those having COVID-19 (*n* = 20), asymptomatic patients tended to be younger (median age 59 vs. 65), had less co-morbidities (median number 1 vs. 2), were less heavily pretreated (median number therapy lines 1 vs. 2 prior SARS-CoV-2) and received less frequent immunotherapy (25% vs. 45%) at the time point of SARS-CoV-2 diagnosis (Table S2).

#### 3.3.2. SARS-CoV-2 Infection with COVID-19 Symptoms

Of the patients with COVID-19, 29% (*n* = 18/63) had a mild, 19% (*n* = 12/63) severe and 20% (*n* = 13/63) critical course. Patients with hematologic malignancies tended to have a critical course more frequently (25%, *n* = 8/32) than those presenting with solid tumors (16%, *n* = 5/31). In contrast, patients with solid tumors more frequently had a severe course of the disease (26%; *n* = 8/31 vs. 13%; *n* = 4/32) (Figure 2). Of note, the risk of a severe or critical COVID-19 course was significantly higher for patients with lymphocytopenia diagnosed within two weeks preceding the SARS-CoV-2 infection or at its presentation (59%) than for those with normal lymphocyte counts (59% vs. 20%, *p* = 0.015). The patients with a severe/critical COVID-19 course had a trend towards worse remission (64% with stable or progressive disease) and general health status (56% with ≥2 co-morbidities). Yet, an advanced age (<60 vs. ≥60) at diagnosis of SARS-CoV-2 correlated significantly with a severe/critical COVID-19 course (*p* = 0.039). The majority (63%) of the patients was treated as inpatients, mostly because of COVID-19 symptoms, but also because they were already in the hospital when SARS-CoV-2 was detected, or were admitted for other indications such as pregnancy or severe lymphodepletion (see Table 3). Of all patients, 28 (44%) remained on the ward, while 12 (19%) had to be admitted to intermediate or intensive care unit (IMC/ICU). Of the latter, nine patients (75%) received respiratory support beyond simple oxygen administration presented either by high-flow oxygen (17%, *n* = 2/12) and non-invasive ventilation (25%, *n* = 3/12), or invasive (mechanical) ventilation (33%, *n* = 4/12). The median length of hospital stay of the patients admitted because of COVID-19 was 14 days (range 1–43). They were treated with a variety of modalities including antivirals, convalescent plasma, dexamethasone, antibodies against IL-6 or IL-6 receptor or serine protease inhibitor (see Table 3).

#### 3.3.3. Outcomes of SARS-CoV-2 Infection with Impact on Cancer Course

**Figure 3:**
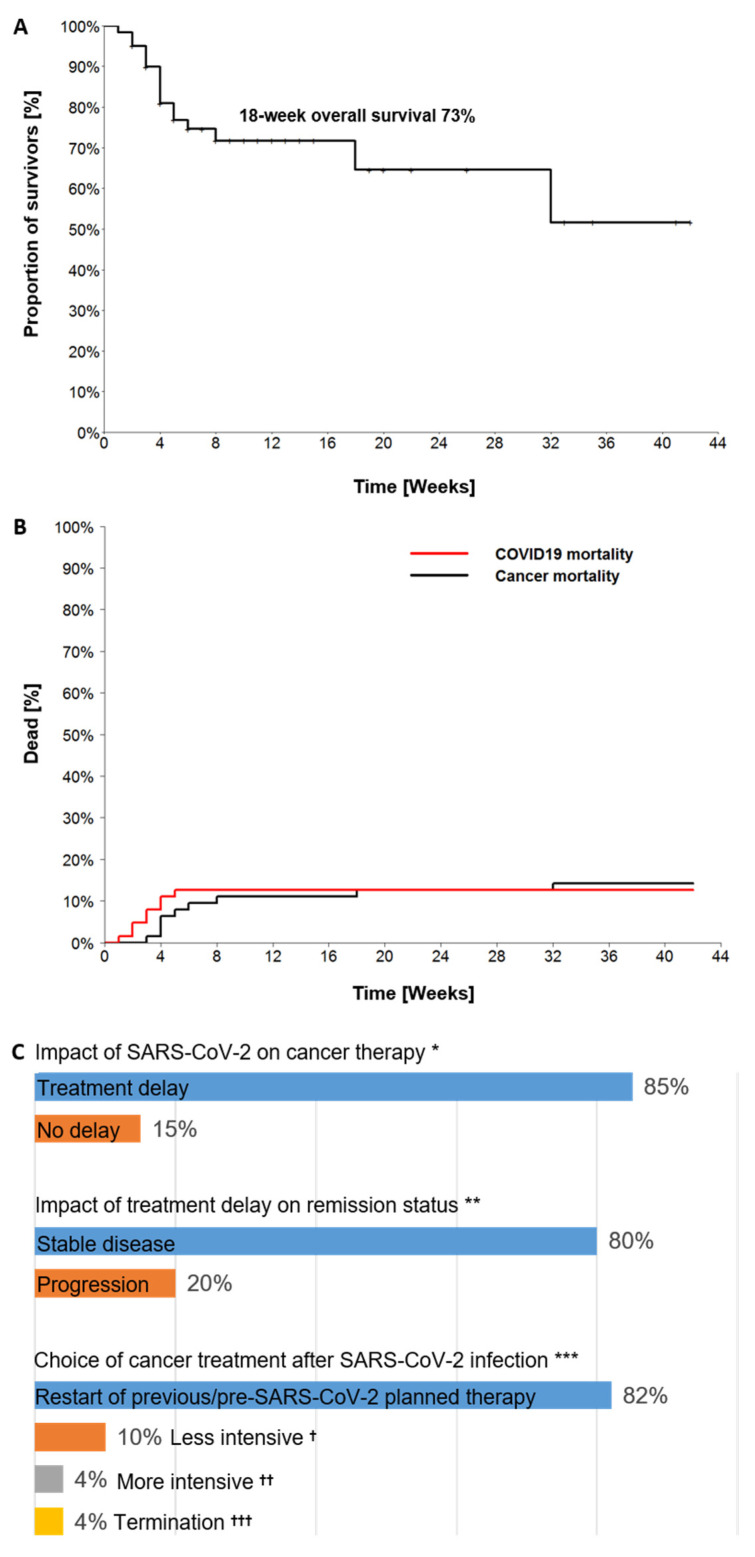
(**A**) Overall survival in the 63 cancer patients with SARS-CoV-2 infection from this study. (**B**) Cumulative percentage of deaths due to COVID-19 and cancer. (**C**) Impact of SARS-CoV-2 infection on the subsequent cancer course. * Results of 54 out of 63 patients with ongoing or planned cancer therapy before testing positive for SARS-CoV-2; 85% of those were observed with delay of cancer therapy. ** Results in 46 patients with treatment delay due to SARS-CoV-2 infection; 80% had stable and 20% progressive disease; *** Illustration of subsequent cancer treatment in 45 SARS-CoV-2 survivors with active malignancy requiring further treatment. ^†^ 10% of these patients received less intensive therapy due to deterioration of general condition following COVID-19; ^††^ 4% received more intensive therapy due to progressive disease following therapy delay after SARS-CoV-2 infection; ^†††^ 4% experienced termination of therapy due to poor general condition following COVID-19.

Outcomes and impact of the SARS-CoV-2 infection on the subsequent cancer course are presented in Table 3 and Figure 3A–C. Median time from SARS-CoV-2 positivity to the last follow-up was 6 weeks (range 0–42). At the time of the last follow-up, 17 patients (27%) had died. Of them, COVID-19 was the cause of death in eight patients (47%) and the malignancy in nine (53%). The calculated four-month overall survival rate (Kaplan-Meier) post-SARS-CoV-2 was 73% (Figure 3A). Of the eight patients that succumbed to COVID-19, four had lung cancer and four a hematologic malignancy: either B-non-Hodgkin lymphoma (B-NHL) (3/8) or acute myeloid leukemia (1/8). Seven of the deceased were men, and the median age was 66 years (range 50–82). Notably, COVID-19 associated deaths occurred within the first month post-SARS-CoV-2, whereas cancer-associated deaths occurred later in the following time (Figure 3B).

Cancer treatment was delayed in 46 (85%) of the 54 patients with ongoing or scheduled cancer therapy prior to their testing positive for SARS-CoV-2 (Figure 3C). The median treatment delay was 25 days (range 4–66). Nine of these patients (20%) developed progressive disease (PD) during the SARS-CoV-2 infection, which may have been associated with the treatment delay. Four of these nine patients later died of the relapsed/progressive disease, and only one of those four had been able to resume cancer therapy. The median treatment delay in these nine patients was 31 days from the positive SARS-CoV-2 test (range 21–54).

Therapy was restarted in 37 of the 45 SARS-CoV-2 survivors with active cancer (82%) and modified for the other eight patients after the SARS-CoV-2 infection. In six patients the therapy was either reduced in intensity (4/6) or terminated (2/6) due to a deterioration of the performance status post-COVID-19. In two other patients more intensive regimens were necessary due to PD developed during the delay in therapy (Figure 3C). At the time of the last follow-up, 20% of the patients (*n* = 13) had CR, 16% (*n* = 10) had PR, 32% (*n* = 20) had SD and 32% (*n* = 20) had relapsed/progressive disease.

## 4. Discussion

Here, we comprehensively analyzed different diagnostic and therapeutic strategies in a cohort of 63 SARS-CoV-2-positive tested cancer patients with the aim of providing real-life data on the management and prognosis of these vulnerable patients.

Our patient cohort encompassed the whole spectrum of solid and hematological neoplasms as well as cancer treatment modalities in outpatient and inpatient care settings. The patients’ characteristics reflected everyday clinical practice as far as more than half of the patients were ≥60 years old, had received ≥1 therapy lines, had predominantly SD or relapsed/progressive disease and had at least on comorbidity. Of note, around half of cancer patients were initially asymptomatic and were diagnosed with SARS-CoV-2 during routine or contact tracing screening. Thirty-three percent of these patients developed COVID-19 after a median interval of approximately two weeks after the first positive SARS-CoV-2 test result. Thus, clinical vigilance together with regular or on-demand testing should be considered to prevent the spread of SARS-CoV-2 among these highly vulnerable patients. Only around two third of the patients tested for SARS-CoV-2 antibodies in our study met the criteria for seroconversion. Several other studies have recently reported a lower level of antibody development in cancer patients [14,15]. Therefore, the risk of SARS-CoV-2 re-infection may be increased in cancer patients, and all established preventive measure should be continued in the post-SARS-CoV-2 follow-up period.

Overall, around 60% of our patients were either asymptomatic or had a mild COVID-19 course. The asymptomatic cases (33%) were not associated with a younger age, a lack of co-morbidities, better remission status, number of ongoing therapies or histologic type of cancer (hematologic vs. solid neoplasms). Indeed, a significant number of these patients were more than 60 years old and presented with relapsed/progressive or stable disease, two or more comorbidities and two or more therapy lines. However, our data is consistent with those of the previous studies, which reported a high incidence of asymptomatic SARS-CoV-2 infections among cancer patients. The incidence of severe and critical courses was 39%, which was comparable with other large multicenter studies in cancer patients with COVID-19 [11,16]. Particularly, patients with hematologic neoplasms were more prone to develop a critical COVID-19 course (25%) which could be explained by a stronger immune dysregulation due to underlying disease and/or applied cancer treatment modalities. Accordingly, a large meta-analysis of 3377 cases with hematologic malignancy and COVID-19 found a 34% risk of death for this patient collective [17]. The most frequently recorded COVID-19 symptoms among our patients were in agreement with those documented for unselected patients [18] and encompassed fever, cough and dyspnea (56%–67%), whereas gastrointestinal complains and chest pain were found in fewer than 15% of the COVID-19 cases.

Particularly, lymphocytopenia preceding SARS-CoV-2 was associated with an almost three-fold increased risk for the development of a severe or critical course compared with cancer patients without lymphocytopenia in our study. This was corresponding to the risk of severe COVID-19 courses in the case of lymphocytopenia in a meta-analysis including 2282 non-cancer SARS-CoV-2 cases [19]. Considering the high frequency of post-treatment lymphodepletion in cancer patients (46% in our study), especially those patients with peri-SARS-CoV-2 lymphocytopenia should be monitored particularly closely. It should be noted that nineteen percent of the patients in our study required admission to IMC/ICU due to COVID-19. For ICU admission, recent studies reported a wide range between 7% and 36% among cancer patients [11,16,20,21]. The ICU admission rate in our cohort was comparable with unselected COVID-19 patients in Germany [22,23]. 75% of all IMC/ICU admitted patients in our study required respiratory therapy beyond standard oxygen administration. Particularly, invasive (mechanical) ventilation was applied for a third of the patients admitted to intensive care whereas second third received non-invasive respiratory support. Notably, the percentage of invasive ventilation was considerably lower than the approximately 54% rate in unselected COVID-19 patients in ICU [23]. Such a difference could be partly explained by the refusal of mechanical ventilation by a medical team due to a poor overall prognosis and/or a patient wish at the time point of COVID-19. In addition, secondary bacterial infection, presented mostly by gram-negative bacteria, accompanied more than third of critical COVID-19 cases in our study and should therefore be seriously considered in diagnostic and treatment workup for cancer patients admitted to ICU. Particularly, Klebsiella pneumoniae was the most common pathogen and documented also commonly in non-cancer patients with critical COVID-19 [24,25].

In line with previous studies [11,12], the overall-case mortality averaged 27% in our cohort with an estimated four month-OS of 73% post-SARS-CoV-2. However, only half of all deaths’ cases were due to COVID-19, with the remaining patients succumbing to progressive malignancy in the course of the SARS-CoV-2 infection. Notably, the deaths from COVID-19 were equally distributed between lung cancer and hematologic malignancy. Eighty-seven percent of the deceased were men. These results were in correspondence with the literature demonstrating the increased risk profile for a severe COVID-19 course of the respective patients [16,26,27,28,29].

Previous studies mainly reported short post-SARS-CoV-2 observation periods of two to three weeks following SARS-CoV-2 detection [11,12,16,20]. The strength of our study was a longer median follow-up (six weeks) post-SARS-CoV-2 with more than 30% patients monitored for 2.5 months or more after the SARS-CoV-2 infection. This enabled us to assess the long-term impact of the SARS-CoV-2 infection on the subsequent cancer management. Indeed, treatment was delayed in 85% of the patients post-SARS-CoV-2 with a median delay time of three and a half weeks. This may have contributed to 20% of the patients developing progressive disease during the delay. Notably, only around four-fifths of the patients were able to resume the previous or pre-SARS-CoV-2 planned therapy, whereas the therapy of the remaining patients was modified, predominantly with a switch to a less intensive regimen or to best-supportive care. Consequently, oncologists face two challenges in the SARS-CoV-2 pandemic: managing SARS-CoV-2 infection with the least possible harm without delaying subsequent cancer management. Careful clinical surveillance together with intensive testing for SARS-CoV-2 should be conducted for the earliest possible re-start of cancer therapy. The role of testing for antibodies to SARS-CoV-2 has not been conclusively answered and requires further investigation. Given the morbidity of COVID-19 and subsequent treatment delay, patients with cancer should be considered high-priority for the COVID-19 vaccination. Considering the efficacy of SARS-CoV-2 vaccine among per se immunocompromised cancer patients, many questions may arise especially for those developing B- and/or T-cell depletion following cancer treatment. As a result, the appropriate time point for vaccination of this patient collective remains undefined. Recently, one study reported that the first dose of a COVID-19 vaccine (either Pfizer-BioNTech or Oxford-AstraZeneca) was able to induce an immune response in around 70% of patients with multiple myeloma. Yet, patients with worse remission status (SD/PD) and suppression of polyclonal (physiologic) immunoglobulins were more likely to lack a seropositive response following a vaccination [30]. Even though the initial clinical studies of the COVID-19 vaccines did not include cancer patients, the European Society for Medical Oncology (ESMO), the Society for Immunotherapy of Cancer (SITC) and the National Comprehensive Cancer Network (NCCN) COVID-19 Vaccination Advisory Committee have recently released their preliminary recommendations supporting vaccination of all patients with cancer, including those receiving active therapy [31,32,33].

Our study has three main limitations. First, our pilot study includes only a relatively small number of patients. Second, the patient group is heterogenic and included different entities and treatment modalities at the time point of enrollment in the study. Third, the data were retrospectively collected from the registries of three cancer centers and patients treated in oncological outpatient clinics are underrepresented.

## 5. Conclusions

Based on our data and the current literature we summarize the following key-points:

1. At diagnosis of SARS-CoV-2 infection in cancer patients, the likelihood of an asymptomatic or mild course of COVID-19 seems to be greater than that of a severe or critical one.
2. Cancer patients diagnosed in an asymptomatic phase of a SARS-CoV-2 infection have around a 30% probability for the subsequent development of COVID-19.
3. Diagnostic and clinical pitfalls such as a lack of seroconversion post-SARS-CoV-2 in some of the cancer patients should be considered when planning the management of SARS-CoV-2 infection and subsequent cancer therapy.
4. Cancer patients with peri-SARS-CoV-2 lymphocytopenia are especially at risk for the development of a severe or critical COVID-19 course and should be monitored carefully.
5. Secondary bacterial infection, presented predominantly by gram-negative bacteria, is common in cancer patients with critical COVID-19 and should be included in a diagnostic and treatment workup for such cases.
6. Even though the COVID-19 mortality is higher in cancer patients as compared to non-cancer individuals, the frequency of ICU admission does not necessarily differ significantly compared with non-cancer COVID-19 patients.
7. A delay in cancer treatment is extremely likely following SARS-CoV-2 infection and requires thorough clinical and diagnostic surveillance to enable for the earliest possible restart of treatment.

## Figures and Tables

**Table 1:** Clinical characteristics of 63 cancer patients at the time of SARS-CoV-2 diagnosis. M, male; F, female; *n*, number; B-NHL, B-cell non-hodgkin lymphoma; T-NHL, T-cell NHL; ALL, acute lymphoblastic leukemia; AML, acute myeloid leukemia; PNH, paroxysmal nocturnal hemoglobinuria.

Parameter	All Patients, *n* = 63	
Gender (M/F), *n* (ratio)	35/28	1.2
Median age, years (range)	62	19–85
Cancer entities, *n* (%)	*n* = 63 (100%)	
Solid tumors, *n* (%)	31/63	(49%)
Lung cancer	8	12%
Breast cancer	7	11%
Gastrointestinal cancer	7	11%
Sarcoma	2	3%
Gynecologic cancer	2	3%
Endocrine cancer	2	3%
Glioma	1	2%
Melanoma	1	2%
Urogenital cancer	1	2%
Hematologic neoplasms, *n* (%)	32/63	(51%)
Lymphoma	22	35%
- B-NHL	20	31%
- T-NHL	1	2%
- Hodgkin lymphoma	1	2%
Multiple myeloma	2	3%
Acute leukemia	7	11%
*- ALL*	4	6%
*- AML*	3	5%
PNH	1	2%
Cancer treatment preceding SARS-CoV-2 positivity, *n* (%)		
Conventional chemotherapy	37	59%
- cytostatic agents only	20	32%
- combined with immunotherapy	10	16%
- combined with targeted therapy	6	9%
- combined with radiotherapy	1	2%
Immunotherapy	7	11%
- immunotherapy monoregimen	5	8%
- combined with radiotherapy	2	3%
Targeted therapy	4	6%
Surgery	4	6%
Hormonal therapy	1	2%
Radiotherapy	1	2%
No therapy yet due to first diagnosis or “watch and wait” strategy	2	3%
Aftercare following systemic cancer treatment	7	11%
Number of therapy lines at SARS-CoV-2 positivity, *n* (%)		
No therapy yet	2	3%
One therapy line	30	48%
Two therapy lines	20	32%
Three therapy lines	3	5%
≥Four therapy lines	8	12%
Remission status at SARS-CoV-2 positivity, *n* (%)		
Complete remission	12	19%
Partial remission	12	19%
Stable disease	11	17%
Relapsed/progressive disease	20	32%
Not yet assessed	8	13%
Comorbidities, *n* (%)		
Cardiovascular disease	37	59%
- arterial hypertension	33	52%
- coronary artery disease	9	14%
- chronic heart failure	5	8%
- atrial fibrillation	3	5%
Diabetes mellitus	9	14%
Metachronous cancer in the history	9	14%
Chronic respiratory disease	7	11%
Chronic kidney failure	7	11%
Obesity	5	8%
Cerebrovascular disease	4	6%
Autoimmune disorders	4	6%
Chronic liver disease	2	3%
No comorbidities	18	29%
Coincidence of comorbidities, *n* (%)		
One comorbidity	16	25%
Two comorbidities	15	24%
Three comorbidities	9	14%
≥Four comorbidities	5	8%

**Table 2:** Laboratory, imaging and clinical findings in 63 cancer patients with SARS-CoV-2. RT-PCR, real-time reverse transcriptase polymerase chain reaction; *n*, number; CT, computed tomography; WBCs, white blood cells; CRP, c-reactive protein; PCT, procalcitonin; LDH, lactate dehydrogenase; pts, patients.

Parameter	All Patients, *n* = 63	
Median time from last cancer treatment to first positive SARS-CoV-2 RT-PCR test, days (range)	13	0–904
Indication for SARS-CoV-2 testing, *n* (%)		
Patients with COVID-19 symptoms	33	52%
Routine or contact tracing screening of asymptomatic patients	30	48%
- subsequent onset of COVID-19 symptoms	10/33	33%
- asymptomatic SARS-CoV-2 during entire follow-up	20/33	67%
- median time from positive SARS-CoV-2 test to onset of COVID-19 symptoms days (range)	13	(6–24)
COVID-19 symptoms (multiple answers possible), *n* (%)	43/63	68%
Fever	29/43	67%
Cough	25/43	58%
Dyspnea	24/43	56%
Gastrointestinal symptoms	6/43	14%
Chest pain	6/43	14%
Laboratory results at SARS-CoV-2 detection		
WBCs × 10^9^/L, median (range) *	4.6	(0.2–12.5)
CRP mg/dl, median (range) **	18.9	(1.0–207)
PCT ng/mL, median (range) ***	0.19	(0.03–2.2)
LDH U/L, median (range) ****	259	(86–1301)
Lymphocytopenia (<1.0 × 10^9^/L) (data available for 37 patients)	17/37	46%
Chest imaging for diagnosing COVID-19, *n* (%) (data available for 39 patients)		
X-ray	24/39	61%
CT	24/39	61%
Ultrasound	3/39	8%
Imaging results, *n* (%)		
Signs of pneumonia	23/39	59%
No indication of pneumonia	16/39	41%
SARS-CoV-2 seroconversion ^†^, *n* (%) (data available for 16 patients)		
- seroconversion achieved	10/16	63%
- median time from SARS-CoV-2 diagnosis by RT-PCR to seroconversion or last negative antibody test, days, range	13	(3 ^††^–133)
Confirmed secondary bacterial infections in COVID-19 cases, *n* (%)	6/43	14%
Detected microorganisms (multiple answers possible), *n* (%)		
Gram-negative bacteria	5/6	83%
- Klebsiella pneumonia	4	67%
- Klebsiella oxytoca	1	17%
- Escherichia coli	2	34%
- Pseudomonas aeriginosa	1	17%
Gram-positive bacteria	1/6	17%
- Staphylococcus aureus	1	17%
Positive specimen/compartment (multiple answers possible), *n* (%)		
- trachealbronchial fluid	4/6	67%
- blood culture	3/6	50%
- urine (including catheter urine)	2/6	33%

* available for 38/63 patients; ** available for 32/63 patients; *** available for 16/63 patients; **** available for 33/63 patients; ^†^ defined as transition of IgG and/or IgA against SARS-CoV-2 from negative to positive result; ^††^ one asymptomatic patient was found to have SARS-CoV-2 antibodies at day 3 following RT-PCR based diagnosis of SARS-CoV-2.

**Table 3:** Outcomes of SARS-CoV-2 infection in 63 cancer patients. *N*, number; IMC, intermediate care; ICU, intensive care unit; PD, progressive disease.

Parameter	All Patients, *n* = 63	
Severity of SARS-CoV-2 infection, *n* (%)		
Asymptomatic SARS-CoV-2 course	20	32%
COVID-19	43	68%
- mild course	18	29%
- severe course	12	19%
- critical course	13	20%
Patient care, *n* (%)		
Outpatients	23	37%
Inpatients	40	63%
- admitted because of COVID-19 symptoms	26	41%
- routine admission prior to positive SARS-CoV-2 test, of them:	12	19%
a. subsequent COVID-19	8	13%
b. asymptomatic SARS-CoV-2	4	6%
- admission of asymptomatic SARS-CoV-2 cases for observation	2	3%
Requiring wards during hospitalization, *n* (%)		
General ward	28	44%
IMC/ICU	12	19%
Non- and invasive respiratory support on IMC/ICU, *n* (%)	9/12	75%
Non-invasive respiratory support	5/12	42%
- high-flow oxygen	2/12	17%
- non-invasive ventilation	3/12	25%
Invasive (mechanical) ventilation	4/12	33%
Median length of hospital stay of patients admitted because of COVID-19, days, range	14	1–43
Treatment modalities related to SARS-CoV-2 infection, *n* (%)		
No therapy	19	30%
Symptomatic therapy only	18	29%
Specific COVID-19 therapy	11	17%
- remdesevir	9	14%
- convalescent plasma	7	11%
- dexamethasone	2	3%
- anti-IL-6 receptor/anti-IL-6 antibody	2	3%
- serine protease inhibitor	1	2%
Antibiotic therapy	19	30%
Treatment delay due to SARS-CoV-2 among 54/63 patients with ongoing or planned cancer therapy before, *n* (%)	46/54	85%
Median time of treatment delay, days, range	25	4–66
Median time from SARS-CoV-2 detection to last follow-up, weeks, range	6	0–42
Remission status at last follow-up, *n* (%)		
Complete remission	13	20%
Partial remission	10	16%
Stable disease	20	32%
Relapse/progressive disease	20	32%
Survival status at last follow-up, *n* (%)		
Alive	46	73%
Dead	17	27%
Causes of death (*n* = 17)		
COVID-19	8	47%
Relapsed/refractory malignancy	9	53%

## Data Availability

The data presented in this study are available on request from the corresponding author.

## Supplementary Materials

The following is available online at https://www.mdpi.com/article/10.3390/cancers13122917/s1, Table S1: clinical characteristics and outcomes in asymptomatic patients with SARS-CoV-2. Table S2: Comparison of asymptomatic and symptomatic SARS-CoV-2 patients with hematologic malignancies.
