# Supplementary material for: Diagnostic, Clinical and Post-SARS-CoV-2 Scenarios in Cancer Patients with SARS-CoV-2: Retrospective Analysis in Three German Cancer Centers
Source: Cancers (Basel). 2021 Jun 11;13(12):2917. doi: 10.3390/cancers13122917 (PMC8230714; doi:10.3390/cancers13122917)
Supplement: Supplementary file 1 [file cancers-13-02917-s001.zip › cancers-1214699-supplementary.pdf]

Table S1. Clinical characteristics and outcomes in asymptomatic patients with SARS-CoV-2. MCL, mantle cell lymphoma; R/R, relapsed/progressive disease; PMBC, primary mediastinal B-cell lymphoma; PR, partial remission; MZL, marginal zone lymphoma; CR, complete remission; SD, stable disease; HDCT/ASCT, high-dose chemotherapy/autologous stem cell transplantation; T-LBL, T-cell lymphoblastic lymphoma; B-ALL, B-cell acute lymphoid leukemia; APL, acute promyelocytic leukemia; T-ALL, T-cell ALL; allo-SCT, allogeneic stem cell transplantation; PNH, paroxysmal nocturnal hemoglobinuria; CTX, chemotherapy.

Patient №	Age	Cancer Type	Comorbidities, №	Prior Lines of Therapy, №	Remission Status at SARS-CoV-2 Positivity	Cancer Therapy at SARS-CoV-2 Positivity	Admission Following SARS-CoV-2 Positivity	Weeks From SARS-CoV-2 Positivity to Last FU	Status at last Follow-Up
					Hematologic neoplasms				
					Lymphomas				
1	66	MCL	3	2	R/R	rituximab/bendamustine/ cytarabine	admission before SARS-CoV-2	5	death in R/R under ibrutinib
2	21	PMBC	0	1	PR	aftercare following R-CHOEP and radiation	no	5	alive in PR, aftercare
3	85	MZL	2	1	PR	rituximab/bendamustine	no	4	alive in CR, ongoing immuno-CTX
4	81	DLBCL	3	5	R/R	rituximab/bendamustine/ polatuzumab vedotin	no	35	alive in CR, aftercare
5	36	Hodgkin lymphoma	0	2	SD	HDCT/ASCT	admission before SARS-CoV-2	6	alive in PR, aftercare
6	61	MCL	0	1	R/R	aftercare in progress	admission before SARS-CoV-2	11	alive in R/R, under ibrutinib

7	34	T-LBL	1	1	CR	aftercare following GMALL 08/2013	no	6	alive in CR, aftercare
						Acute leukemias			
8	59	B-ALL	1	2	CR	maintenance, GMALL 08/2013	no	13	alive in CR, aftercare
9	73	APL	3	1	CR	arsenic trioxide/ all-trans retinoic acid	no	9	alive in CR, ongoing therapy
10	21	T-ALL	0	1	CR	induction, GMALL 08/2013	no	15	alive in CR, post-allo-SCT
						Others			
11	71	Multiple myeloma	4	1	PR	dexamethasone/ lenalidomide	No	4	alive in PR, ongoing therapy
12	36	PNH with pregnancy	0	1	SD	eculizumab	admission for observation	5	alive, stable PNH and pregnancy
						Solid neoplasms			
						Gastrointestinal cancer			
13	62	Liver cancer	4	1	SD	surgery	admission before SARS-CoV-2	22	alive in PR, post-CTX
14	62	Liver cancer	2	2	R/R	gemcitabine/ cisplatin	admission before SARS-CoV-2	7	alive in R/R, ongoing CTX
15	66	Pancreatic cancer	2	1	SD	FOLFIRINOX	no	8	alive in SD, ongoing CTX
16	69	Colon cancer	1	2	SD	5-fluorouracil/ bevacizumab	no	9	alive in R/R, change to FOLFOX
						Sarcomas			
17	42	Sarcoma	0	3	PR	dacarbazine/L19TNF-PH-L19TNFSARC-08/18	no	5	alive in R/R, ongoing CTX

18	19	Sarcoma	0	1	SD	doxorubicin/Ifosfamide	admission for observation	2	alive in SD, CTX restart planned
Others									
19	75	Breast cancer	2	7	R/R	faslodex/letrozol	no	13	alive in R/R, palliative care
20	64	Lung Cancer	1	2	PR	pemetrexed/ pembrolizumab	no	4	alive in CR, aftercare

Table S2. Comparison of asymptomatic and symptomatic SARS-CoV-2 patients with hematologic malignancies. CR, complete remission; PR, partial remission; SD, stable disease; PD, progressive disease.

**Categories**	**COVID-19 Patients with Hematologic Malignancies (*n* = 20)**	**Asymptomatic SARS-CoV-2 Patients with Hematologic Malignancies (*n* = 12)**
Entities, %	B-lymphoid neoplasms 90%<br>myeloid neoplasms 10%	B-lymphoid neoplasms 84%<br>myeloid neoplasms 16%
Age, median (range)	65 (27–82)	59 (21–81)
Gender, male:female (%)	60%:40%	50%:50%
Co-morbidities, number, median (range)	2 (0–3)	1 (0–4)
Remission status at SARS-CoV-2 diagnosis	CR 35%<br>PR 10%<br>SD 10%<br>relapsed/PD 30%<br>not assessed yet 15%	CR 33%<br>PR 25%<br>SD 17%<br>relapsed/PD 25%
Days from last cancer treatment to SARS-CoV-2 diagnosis, median (range)	22 (0–904)	11 (1–199)
Therapy lines, number, median (range)	2 (0–5)	1 (1–5)
Cancer therapy at diagnosis of SARS-CoV-2 (%)	follow-up surveillance 15%<br>conventional chemotherapy 20%<br>immunotherapy 45%:<br>- with chemotherapy 25%<br>- immunotherapy alone 15%<br>- with radiotherapy 5%<br>targeted therapy 10%<br>radiotherapy 5%<br>no therapy yet (planned) 5%	follow-up surveillance 25%<br>conventional chemotherapy 50%<br>immunotherapy combined with chemotherapy 25%
